# A Streamlined One-Pot Extraction Method Enables High-Sensitivity Top-Down Proteomic Characterization of Phospholamban from Biopsy-Scale Cardiac Tissue

**DOI:** 10.1021/jasms.6c00309

**Published:** 2026-09-19

**Authors:** Isabella R. Clemmer, Holden T. Rogers, Mallory C. Wilson, Zhan Gao, Hsin-Ju Chan, Zachery R. Gregorich, Farhan Raza, Ying Ge

**Affiliations:** † Department of Chemistry, 5228 University of Wisconsin-Madison, Madison, Wisconsin 53706, United States; ‡ Department of Cell and Regenerative Biology, University of Wisconsin-Madison, Madison, Wisconsin 53705, United States; § Department of Animal and Dairy Sciences, University of Wisconsin-Madison, Madison, Wisconsin 53706, United States; ∥ Department of Medicine-Cardiovascular Disease, University of Wisconsin-Madison, Madison, Wisconsin 53705, United States; ⊥ Cardiovascular Research Center, School of Medicine and Public Health, University of Wisconsin-Madison, Madison, Wisconsin 53706, United States; # Human Proteomics Program, School of Medicine and Public Health, University of Wisconsin-Madison, Madison, Wisconsin 53706, United States

## Abstract

Top-down proteomics (TDP) enables comprehensive characterization of intact proteoforms, but the analysis of membrane proteoforms remains challenging because of their hydrophobicity and relatively low abundance. To address these challenges, we previously developed Azo, a photocleavable surfactant that enables efficient solubilization and top-down analysis of membrane proteins. Subsequently, we established an Azo-enabled TDP method permitting the comprehensive characterization of phospholamban (PLN), a transmembrane protein that plays crucial roles in calcium handling, directly from cardiac tissue. However, the original workflow required at least 10 mg of human cardiac tissue, making it unsuitable for biopsy-scale and other sample-limited studies. Herein, we developed a streamlined Azo-enabled one-pot extraction strategy for high-sensitivity top-down analysis of PLN that reduces the required tissue input to 1 mg while preserving analytical performance. Using online liquid chromatography–tandem mass spectrometry, we achieved highly reproducible detection, quantification, and post-translational modification localization of endogenous PLN proteoforms from 1 mg of human cardiac tissue. Notably, the streamlined strategy reduced the tissue required for PLN protein extraction 10-fold while retaining the same capacity for comprehensive characterization in the one-pot extraction. Collectively, these results establish a practical strategy for TDP of PLN proteoforms from biopsy-scale and other sample-limited myocardial specimens.

## Graphical abstract

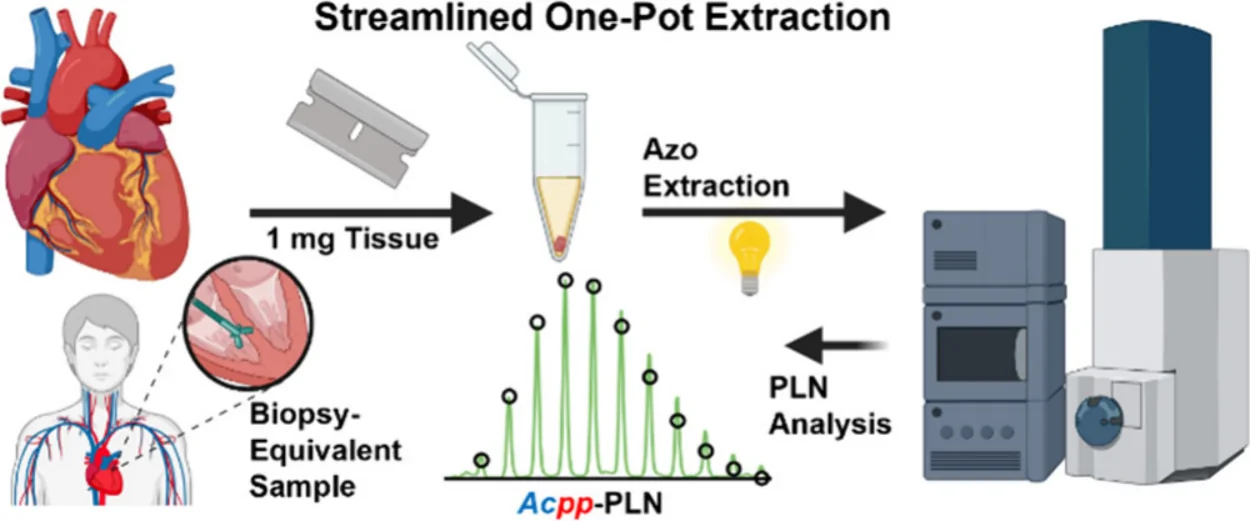

## Introduction

Top-down proteomics (TDP) directly analyzes intact proteins, enabling comprehensive characterization of proteoforms^1,2^ by preserving the molecular connectivity among post-translational modifications (PTMs), genetic variants, and alternative splice isoforms.^3–8^ This unique capability provides insights into proteoform biology, protein function, and disease-associated molecular mechanisms that cannot be readily resolved using peptide-centric approaches.^3,9^ Membrane proteins play critical roles in cellular signaling, transport, and communication across biological membranes, making them important drug targets.^10,11^ However, analysis of intact membrane proteins is particularly challenging due to their hydrophobicity, low abundance, and reduced ionization efficiency.^12–18^

To address these challenges, our laboratory developed a photocleavable surfactant, Azo, enabling efficient solubilization and top-down analysis of membrane proteins.^19,20^ Using Azo, we subsequently established a TDP workflow for comprehensive proteoform characterization of phospholamban (PLN),^21^ a 52-amino-acid transmembrane regulator of sarco/endoplasmic reticulum calcium ATPase 2a (SERCA2A) in the heart.^22–28^ PLN plays a central role in regulating cardiac calcium homeostasis, and its activity is dynamically regulated by PTMs including phosphorylation and S-palmitoylation.^29,30^ Dysregulation of PLN has been implicated in the pathogenesis of heart failure and inherited cardiomyopathies with prominent arrhythmias.^31–34^ The previously established workflow employed a two-stage extraction strategy consisting of cytosolic depletion followed by membrane protein solubilization in Azo surfactant buffer, enabling identification, quantification, and site-specific localization of PLN PTMs.^21^ However, it required a minimum of 10 mg of tissue due to sample loss over multiple extraction and cleanup steps, making it unsuitable for biopsy-scale and other sample-limited studies.

These limitations highlight the need for simplified sample preparation strategies that minimize sample loss and enable top-down proteomic analysis from a limited biological material. One-pot sample preparation strategies have increasingly been developed to reduce sample handling and protein losses, thereby improving the sample efficiency for limited-input proteomics. In particular, the nanodroplet processing in a one-pot trace sample platform (nanoPOTS) has been adapted for top-down proteomics, enabling intact proteoform analysis from low numbers of mammalian cells by minimizing sample loss during sample preparation.^35^ Similarly, other one-pot approaches have incorporated magnetic-bead-based protein capture and intact proteoform elution for low-input top-down analysis.^36^ In our laboratory, one-pot processing has also been applied to single-muscle-cell top-down proteomics to improve the recovery of intact proteoforms.^37^ Collectively, these studies demonstrate the potential of one-pot strategies to reduce sample loss and extend top-down proteomics to increasingly sample-limited biological systems.

Building on these advances, we developed a streamlined azo-enabled one-pot extraction strategy for high-sensitivity top-down analysis of PLN that reduces the required tissue input from 10 mg to 1 mg of cardiac tissue. The streamlined one-pot extraction strategy eliminates the cytosolic depletion step and incorporates sonication-assisted extraction in a photocleavable Azo surfactant buffer to minimize sample loss. Using this strategy, we demonstrate reproducible detection, identification, quantification, and comprehensive characterization of PLN, including phosphorylated and palmitoylated PLN proteoforms, from biopsy-scale tissue inputs with analytical performance comparable to that of the conventional workflow. This work establishes a practical strategy for the biopsy-compatible TDP interrogation of PLN proteoforms.

## Materials and Methods

### Reagents and Chemicals

The reagents used were purchased from MilliporeSigma (Burlington, MA, USA), unless otherwise noted. Buffers contained liquid chromatography (LC)-mass spectrometry (MS)-grade water from Milli-Q (Millipore, Corp., Billerica, MA, USA). Isopropanol (IPA), acetonitrile (ACN), and methanol (MeOH) were obtained from Fisher Scientific (Fair Lawn, NJ, USA).

### Human Cardiac Tissue Collection

Left-ventricular myocardial tissue from nonfailing adult donors was obtained from the University of Wisconsin (UW)-Madison Organ and Tissue Donation Program. The Institutional Review Board (IRBs) of UW-Madison (study no. 2013–1264) approved the collection procedures for human heart tissue.

### Protein Extraction and Sample Preparation

To compare the streamlined one-pot extraction method with the previously established two-stage extraction method,^21^ both protocols were performed in parallel using cardiac tissue from nonfailing donor hearts, with all extraction procedures conducted at 4 °C. For the streamlined one-pot method, cardiac tissues from nonfailing donors were cut into 1 mg pieces, approximating biopsy-scale tissue samples. Unlike the previously established workflow for 10 mg of cardiac tissue, these samples were not cryopulverized and did not undergo cytosolic depletion. Instead, the tissue was immediately homogenized with a Teflon pestle in 100 μL of Azo buffer [25 mM ammonium bicarbonate (ABC)] pH 8.0, 0.4% Azo in 25 mM ABC, 10 mM l-methionine, 5 mM tris­(2-carboxyethyl) phosphine hydrochloride (TCEP), 1 mM phenylmethylsulfonyl fluoride (PMSF), 2.5 mM ethylenediaminetetraacetic acid (EDTA), and 1× Halt protease and phosphatase inhibitor cocktail (Thermo Scientific; Catalog # 78444)] and vortexed vigorously. To compensate for the lack of cryopulverization and ensure efficient protein solubilization, samples were then sonicated in a water bath located in a cold room (4 °C) for 10 min. The homogenate was centrifuged (21,000 *g* for 25 min, 4 °C), and the supernatant was removed (Figure 1).

**Figure 1:**
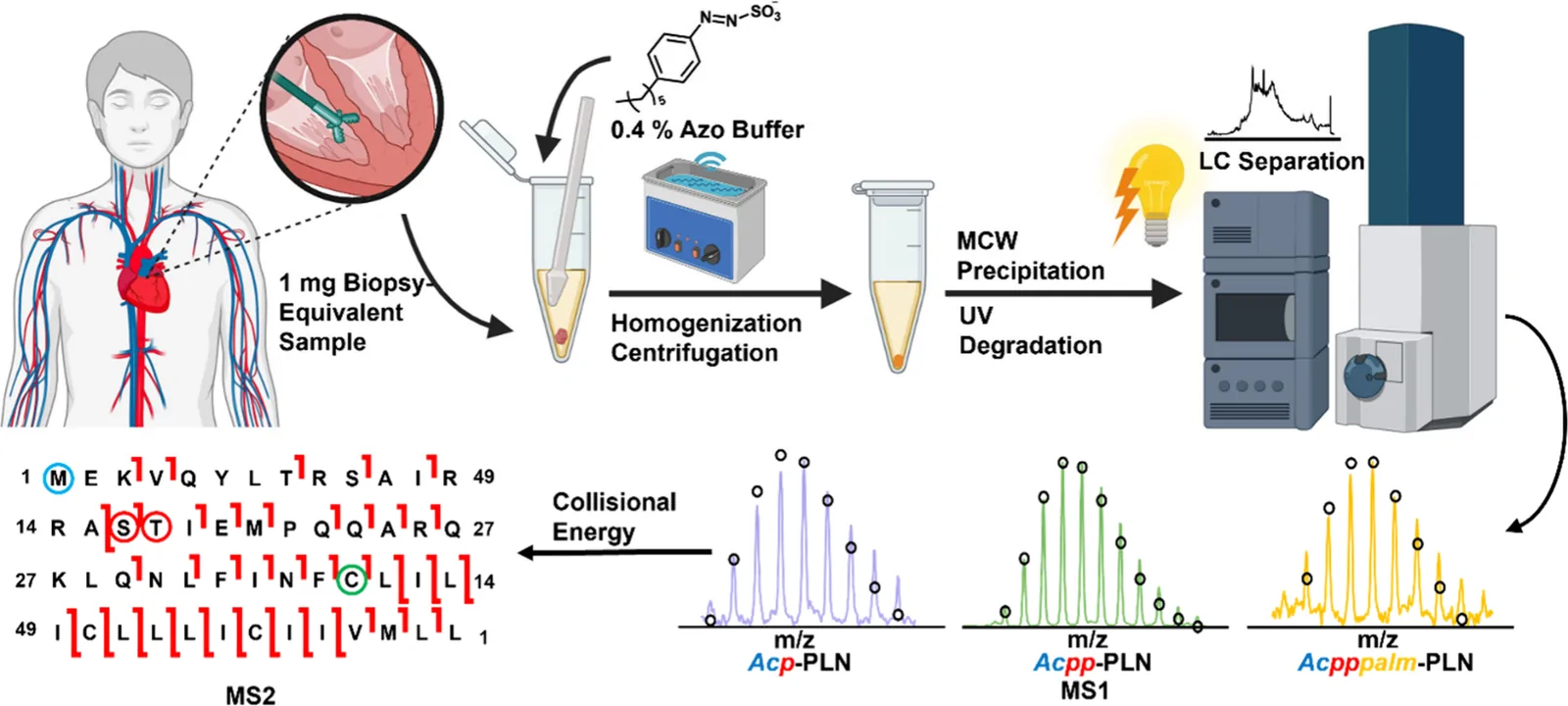
Schematic of the streamlined Azo-enabled one-pot extraction and high-sensitivity TDP workflow from biopsy-scale (1 mg) of cardiac tissue. Cardiac tissue was homogenized in an Azo buffer, followed by a MCW precipitation. The protein pellets were resolubilized, Azo was degraded by UV irradiation, and intact proteins were analyzed by LC-MS/MS using a Bruker QTOF mass spectrometer.

For the previously established two-stage extraction method,^21^ 10 mg pieces of cardiac tissue were cut and cryopulverized under liquid nitrogen. Cytosolic proteins were depleted twice by being homogenized with a Teflon pestle in 100 μL of ABC buffer [25 mM ABC pH 8.0, 10 mM l-methionine, 5 mM TCEP, 1 mM PMSF, 2.5 mM EDTA, and 1× Halt protease and phosphatase inhibitor cocktail]. After centrifuging (21,000 *g* for 25 min, 4 °C), samples were homogenized in 100 μL Azo buffer, and centrifuged again (21,000 *g* for 25 min, 4 °C). The supernatant was then centrifuged one additional time to pellet any cell debris (Figure S1).

For both methods, proteins were prepared for MS analysis using a MeOH/chloroform/water (MCW) precipitation as described previously^21^ (Supplemental Methods). Protein pellets were then resolubilized in 80% formic acid in water and diluted with 10:10:80 IPA:ACN:water before being irradiated with ultraviolet (UV) light for 1 min to degrade any remaining Azo. All extraction steps for both protocols were performed at 4 °C.

### Top-Down LC-Tandem MS (MS/MS) Analysis

LC-MS/MS analysis was performed as previously described,^21^ with the same parameters for samples prepared from the 1 mg and 10 mg extraction analysis. Briefly, protein samples were normalized to a concentration of 0.5 mg/mL and 4 μL (2 μg of total protein) was injected into the system. Samples were analyzed using reversed-phase LC (RPLC) on a Waters NanoAcquity ultraperformance LC system coupled to an Impact II quadruple time-of-flight (QTOF) mass spectrometer (Bruker Daltonics). The RPLC method includes 0.2% formic acid in water for mobile phase A (MPA) and 0.2% formic acid in IPA/ACN for mobile phase B (MPB). A 1 h gradient was performed, going from 10% to 90% MPB (0–5 min, 10% B; 5–45 min, 15%–65% B; 45–50 min, 65%–90% B, 50–55 min, 90% B; 55–60 min, 10% B) on a home-packed column (C2, 150 × 0.5 mm, 5-μm particle size, 300 Å pore size). A constant flow rate of 20 μL/min was used, and the column temperature was set at 60 °C. MS parameters can be found in the Supporting Information (Supplemental Methods). MS1 was performed for intact analysis, and targeted MS/MS was performed for selected PLN proteoforms with collisional activated dissociation (CAD) set between 20 and 50 eV on additional injection replicates. The instrument was tested for reliability and reproducibility using injection replicates, and a loading curve was run to demonstrate that the amount of sample injected was within the linear response range of the instrument (Figure S2).

### Data Analysis

LC-MS/MS data was analyzed with Bruker Daltonics Data Analysis software (ver. 4.3). Extracted ion chromatograms (EIC) were generated from the protein’s most abundant proteoforms’ charge state values to determine elution times of each proteoform and mass spectra were generated by averaging across the total ion chromatogram (TIC) during those elution times. Mass spectra were deconvoluted using Maximum Entropy set at a resolution of 60,000 and relative abundances were calculated using intensity values with the sophisticated numerical annotation procedure (SNAP) peak-picking algorithm with the quality factor threshold set at 0.4, the absolute intensity threshold at 300, and the max charge state at 20. Total phosphorylation levels (Pi/mol protein) of PLN were calculated as described previously^21^ (Supplemental Methods). MASH Native^38^ (Version 1.1) was used to analyze tandem mass spectra from targeted MS/MS. An error tolerance of 20 ppm was used for fragment verification.

### Statistical Analysis

Statistical comparisons of proteoform levels between the 1 mg and 10 mg samples were performed using a Mann–Whitney U test. All *p*-values less than 0.05 were considered statistically significant.

## Results and Discussion

### High-Sensitivity TDP Enables Reproducible Detection of PLN Proteoforms from Biopsy-Scale Cardiac Tissue

To enable proteoform analysis from biopsy-scale cardiac tissue, we developed a streamlined one-pot Azo-enabled extraction method for high-sensitivity TDP (Figure 1). During initial method development, different tissue input amounts (10, 5, 2, and 1 mg) were evaluated to determine the feasibility of reducing the sample requirement while maintaining robust PLN proteoform characterization. Based on these experiments, 1 mg, the lowest tissue input evaluated in this study, was selected for the streamlined one-pot extraction method to enable biopsy-scale analysis and further evaluated in a side-by-side comparison with the conventional two-stage extraction method. In contrast to our previously established conventional two-stage extraction method (Figure S1),^21^ this one-pot extraction method eliminates cryopulverization and cytosolic depletion and incorporates direct sonication in Azo buffer, minimizing sample loss while maintaining robust PLN extraction and reproducibility. The streamlined one-pot extraction method yielded approximately 30–60 μg of total protein from 1 mg of cardiac tissue (1–2 μg/μL in a total extract volume of 30 μL). Left ventricular tissue from a nonfailing donor heart was analyzed using both the newly developed one-pot extraction method (1 mg tissue) and the conventional two-stage extraction method (10 mg tissue), with all extractions and analyses performed in parallel for direct comparison. Each method was performed in triplicate. The TICs acquired using the 1 h elution gradient were highly reproducible for both methods (Figure S3). The TICs differed because the conventional two-stage extraction depleted cytosolic proteins (Figure 2A, C), however both extraction methods reproducibly detected comparable PLN proteoform profiles (Figure 2E, F).

**Figure 2:**
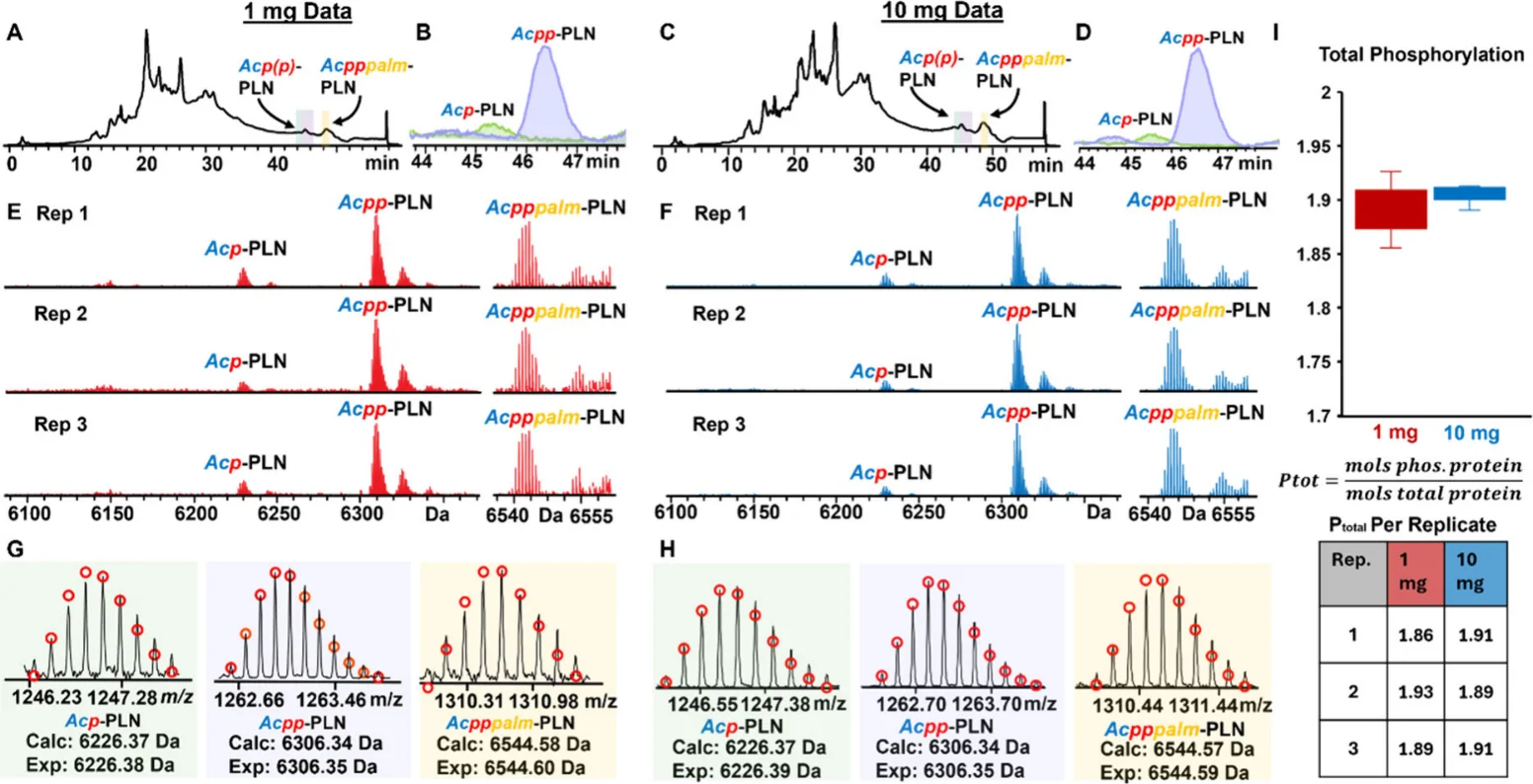
Comparison of PLN proteoform analysis using the streamlined one-pot (1 mg) and conventional two-stage (10 mg) extraction methods. (A, B) TIC and EIC for the one-pot extraction method (1 mg tissue). (C, D) TIC and EIC from the conventional two-stage extraction method (10 mg tissue). (E, F) Mass spectra from three replicates of the one-pot (E) and conventional two-stage (F) extraction methods, showing reproducible detection of PLN proteoforms. (G, H) Representative high-resolution, zoomed-in mass spectra of PLN proteoforms from the one-pot (1 mg) (G) and two-stage (10 mg) (H) extraction methods, including mono- and bis-phosphorylated and palmitoylated bis-phosphorylated forms, with isotopic fitting and monoisotopic masses. (I) Comparison of total phosphorylation between the one-pot (1 mg) and two-stage (10 mg) extraction methods.

EICs revealed reproducible elution of mono- and bis-phosphorylated PLN proteoforms between 45.0 and 47.5 min (Figures 2 and S4), whereas the palmitoylated bis-phosphorylated proteoform eluted between 49.2 and 49.7 min (data not shown). High-resolution mass spectra confirmed the presence of previously reported PLN proteoforms^21^ through mass shifts corresponding to N-terminal acetylation (+42.01 Da), phosphorylation (+79.96 Da), and S-palmitoylation (+238.23 Da).

Importantly, deconvoluted spectra demonstrated highly similar PLN proteoform profiles between the one-pot extraction (1 mg) and the conventional two-stage extraction (10 mg) methods (Figure 2E, F), indicating that the 10-fold reduction in tissue input did not result in observable loss of proteoform diversity or spectral quality. Furthermore, all detected PLN proteoforms were measured within 3 ppm of their theoretical masses in both workflows (Figure 2G, H), demonstrating excellent mass accuracy despite a likely reduction in PLN recovery due to the lack of cytosolic protein depletion. Although the streamlined one-pot extraction method incorporated a 10 min sonication step at 4 °C to facilitate tissue disruption and protein extraction, we did not observe detectable changes in the PLN proteoform distribution or PTM characterization attributable to the sonication treatment under the conditions used in this study. Furthermore, we observed no evidence of acid-induced modifications, as no additional or unexpected modifications consistent with formic acid-induced artifacts were detected. These results demonstrate that the streamlined one-pot extraction strategy enables reproducible detection of low-abundance PLN proteoforms from biopsy-scale cardiac tissue with a 10-fold reduction in tissue input.

### Quantitative Assessment of PLN Phosphorylation Is Preserved Following a 10-fold Reduction in Tissue Input

Having established reproducible detection of PLN proteoforms using the streamlined one-pot extraction method, we next evaluated whether the reduced tissue input affected quantitative measurements. The total PLN phosphorylation levels were calculated from deconvoluted mass spectra. The one-pot extraction method (1 mg tissue) yielded 1.89 ± 0.03 Pi/mol protein, compared with 1.91 ± 0.02 Pi/mol protein for the conventional two-stage extraction method (10 mg tissue) (Figure 2I). No statistically significant difference was observed between the two extraction methods (Mann–Whitney U test, *p* = 0.9999), suggesting that the streamlined one-pot extraction method provides comparable quantitative measurements of PLN phosphorylation. The comparable phosphorylation levels observed between these two extraction methods indicate that reducing tissue input in the streamlined one-pot extraction method preserves the biological information required for PLN proteoform analysis, which is particularly important for the detection of conditions where PLN phosphorylation is known to change such as heart failure and certain genetic diseases.^39–43^ Together, these results demonstrate that quantitative assessment of PLN phosphorylation can be reliably achieved from only 1 mg of human cardiac tissue, expanding the applicability of TDP to sample-limited studies.

### The Streamlined One-Pot Extraction Method Enables High-Confidence PTM Localization

Although the same amount of total protein (2 μg) was injected for both workflows, the composition of the injected samples differed. The conventional two-stage extraction method (10 mg) included cytosolic depletion, enriching membrane proteins such as PLN, whereas the one-pot extraction (1 mg) workflow retained the full proteome. Consequently, the relative amount of PLN injected was expected to be lower in the one-pot workflow due to increased contribution from abundant cytosolic proteins. To determine whether this reduced amount affected MS/MS-based proteoform characterization, mono- and bis-phosphorylated PLN proteoforms were subjected to targeted CAD-MS/MS using collision energies of 20, 30, 40, and 50 eV. Fragment ions from all collision energies were combined to maximize sequence coverage (Figures 3 and S5).

**Figure 3:**
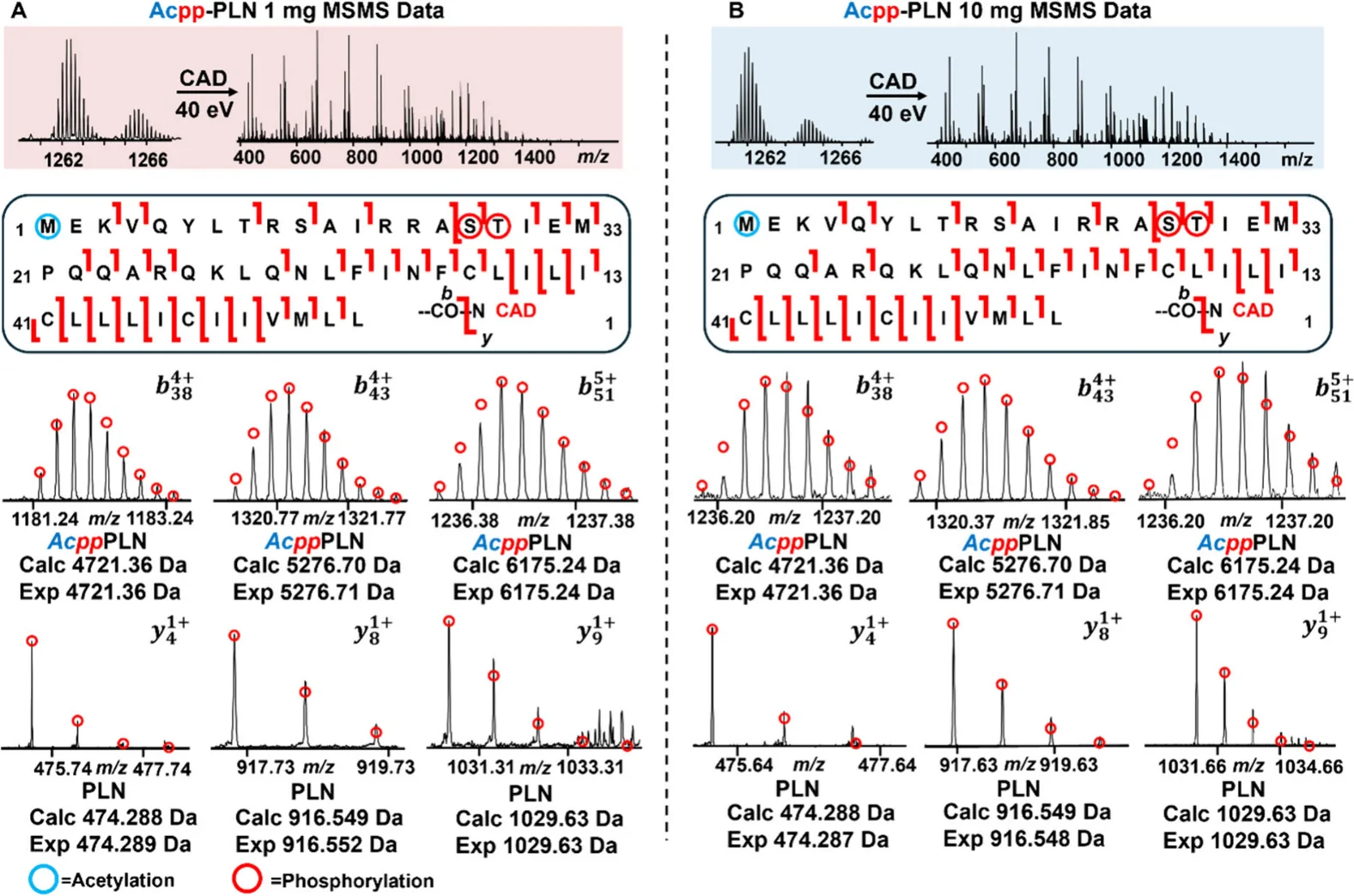
Online LC-MS/MS characterization of bis-phosphorylated PLN proteoform following the streamlined one-pot (1 mg) and conventional two-stage (10 mg) extraction methods. (A) Representative tandem mass spectra acquired at 40 eV of collisional energy following the one-pot extraction (1 mg tissue). Combined fragment ions from CAD at 20, 30, 40, 50 eV collisional energy yielded 49 fragment ions, 36 bond cleavages, and 71.2% sequence coverage. (B) Representative tandem mass spectra of the bis-phosphorylated PLN proteoform acquired at 40 eV of collisional energy following the conventional two-stage extraction (10 mg tissue). Combined fragment ions of 20, 30, 40, 50 eV collisional energy yielded 48 fragment ions, 36 bond cleavages, and 71.2% sequence coverage.

For the bis-phosphorylated proteoform, 49 fragment ions (36 b-ions and 13 y-ions) were identified following the streamlined one-pot extraction method (1 mg tissue), compared with 48 fragment ions (36 b-ions and 12 y-ions) following the conventional two-stage extraction method (10 mg tissue). Both extraction methods achieved identical sequence coverage of 71.2% with 36 bond cleavages out of 52 possible backbone cleavages (Figure 3). For the monophosphorylated proteoform, 43 fragment ions with 33 bond cleavages were identified following the one-pot extraction method, compared with 47 fragment ions and 32 bond cleaves following the conventional two-stage extraction method. The corresponding sequence coverages were 63.5% and 61.5%, respectively (Figure S5). Importantly, preservation of sequence coverage enabled confident localization of phosphorylation sites to Ser16 and Thr17, consistent with previous studies.^22,29,44^ These results demonstrate that the substantial reduction in tissue input in the streamlined one-pot extraction method does not diminish confidence in PTM localization or proteoform identification. Although CAD enabled confident localization of the PLN phosphorylation sites in the present study, electron-based fragmentation methods or ultraviolet photodissociation (UVPD) could potentially improve the preservation and characterization of labile PTMs.^45–50^ The S-palmitoylated PLN proteoform was assigned based on its intact MS1 mass and chromatographic elution rather than targeted MS/MS characterization. Importantly, the site of PLN S-palmitoylation has previously been localized to Cys36 using targeted MS/MS and a range of collision energies in our previous report.^21^ Thus, the present study focused on detection of the S-palmitoylated proteoform by intact mass rather than relocalizing the modification site.

Furthermore, the present study was designed to evaluate the streamlined one-pot extraction method through targeted PLN proteoform analysis rather than global top-down proteomic profiling; therefore, we did not perform comprehensive proteoform identification or determine the total number of proteoforms detected. Given the demonstrated utility of Azo for membrane protein solubilization in our previous studies,^19,20^ we anticipate that the streamlined one-pot method may be applicable to additional membrane protein targets. Future studies will systematically evaluate its applicability to other membrane proteins and cardiac protein classes, including myofilament, mitochondrial, and histone proteins, and assess its overall proteoform coverage.

Collectively, these results establish that the streamlined one-pot extraction method enables comprehensive top-down PLN proteoform analysis from biopsy-scale tissue inputs. Endomyocardial biopsies provide a unique opportunity to provide direct access to myocardial tissue for diagnosis, investigate molecular changes during disease progression, and/or in response to therapeutic interventions.^51,52^ However, their limited tissue yield has historically constrained comprehensive proteoform analysis. Consequently, most previous studies of heart failure rely on tissue obtained at end-stage disease, where distinct pathogenic mechanisms have often converged on common remodeling pathways.^53–56^ By reducing tissue requirements to 1 mg, the present method expands the feasibility of longitudinal and patient-specific top-down proteomic studies using endomyocardial biopsies to identify early proteoform alterations and monitor molecular responses to therapeutic interventions to therapy.

## Conclusion

In this study, we developed a streamlined one-pot extraction method for high-sensitivity TDP that enables reproducible detection and quantification of PLN proteoforms from only 1 mg of human cardiac tissue. By implementing the Azo-enabled one-pot extraction strategy with online RPLC-MS/MS analysis, we achieved analytical performance comparable to our previously established two-stage extraction method with a 10-fold reduction in tissue requirements. The ability to reproducibly detect and comprehensively characterize PLN proteoforms from biopsy-scale tissue inputs substantially improves sample efficiency while preserving valuable clinical specimens. These advances improve the feasibility of sensitive PLN proteoform characterization from limited cardiac tissue inputs and provide a foundation for future studies investigating proteoform remodeling in cardiovascular diseases.

## Supplementary Material



## Notes

Sample preparation and cleanup methods; QTOF instrument parameters; total phosphorylation equation; two-stage extraction schematic; TICs and loading curve from three injection replicates for both the two-stage and one-pot extraction methods; TICs from three extraction replicates for the two-stage and one-pot extraction methods; EICs for mono and bis-phosphorylated PLN for the two-stage and one-pot extraction methods; MS/MS spectra of monophosphorylated PLN with sequence map (PDF)
