# Supplementary material for: A Streamlined One-Pot Extraction Method Enables High-Sensitivity Top-Down Proteomic Characterization of Phospholamban from Biopsy-Scale Cardiac Tissue
Source: J Am Soc Mass Spectrom. 2026 Sep 19;37(10):2273–80. doi: 10.1021/jasms.6c00309 (PMC13651618; doi:10.1021/jasms.6c00309)
Supplement: Supplementary file 1 [file js6c00309_si_001.pdf]

Supporting Information

A Streamlined One-Pot Extraction Method Enables High-Sensitivity Top-Down Proteomic Characterization of Phospholamban from Biopsy-Scale Cardiac Tissue

Isabella R. Clemmer[1], Holden T. Rogers[1], Mallory C. Wilson[1], Zhan Gao[2], Hsin-Ju Chan[1], Zachery R. Gregorich[2,3], Farhan Raza[4,5], and Ying Ge[1,2,5,6,*]

[1] Department of Chemistry, University of Wisconsin-Madison, Madison, Wisconsin, 53706, USA

[2] Department of Cell and Regenerative Biology, University of Wisconsin-Madison, Madison, Wisconsin 53705, USA

[3] Department of Animal and Dairy Sciences, University of Wisconsin-Madison, Madison, Wisconsin 53706, USA

[4] Department of Medicine-Cardiovascular Disease, University of Wisconsin-Madison, Madison, Wisconsin 53705, USA

[5] Cardiovascular Research Center, School of Medicine and Public Health, University of Wisconsin-Madison, Madison, Wisconsin 53706, USA

[6] Human Proteomics Program, School of Medicine and Public Health, University of Wisconsin-Madison, Madison, Wisconsin 53706, USA

* Correspondence may be addressed to Ying Ge, (ying.ge@wisc.edu; 1111 Highland Ave., Room 8551 WIMR II, Madison, WI 53705)

Supplemental Methods

Sample Preparation (Clean-up steps)

For both streamlined one-pot (1 mg) and conventional two-stage (10 mg) extraction methods, protein extracts were cleaned-up and prepared for MS injection using a methanol (MeOH)/chloroform/water (MCW) precipitation method. To begin a MCW precipitation, 300 µL of cold water (4 °C) was added to 100 µL of protein extract and the solution was vortexed. Next, 400 µL of cold MeOH (-20 °C) and 100 µL of cold chloroform (-20 °C) were added with vertexing after each step. The solution was centrifuged (10 min, 18,000 *g*, 4 °C) and a protein pellet formed at the interface. The upper layer was removed, and 400 µL of cold MeOH (-20 °C) was added. The solution was vortexed and centrifuged (10 min, 18,000 *g*, 4 °C) again. This step was repeated twice. Protein pellets were solubilized using 80% formic acid in water (-20 °C), diluted to 5% formic acid with 10:10:80 isopropanol:acetonitrile:water, and placed under ultraviolet light for irradiation for around one min. The samples were centrifuged at max speed 21,000 *g*, 4 °C for 10 min and normalized to 0.5 µg/µL using a Bradford assay.

Impact II Quadruple Time-of-Flight (QTOF) Mass Spectrometer Parameters

All parameters were unchanged between samples. Within the electrospray ionization source, Capillary, Nebulizer, End Plate Offset, Dry Gas, and Dry Temp were set to 4500 V, 0.6 Bar, 500 V, 4.5 L/min and 220 ºC. The optimal tune settings were Ion Energy, Hexapole RF, Funnel 1 RF, Funnel 2 RF, isCID Energy, Transfer Time, Collision Energy, Collision RF, Pre-Pulse Storage set to 600 Vpp, 350 Vpp, 350 Vpp, 350 Vpp, 10 eV, 110 µs, 10 eV, 2600 Vpp, 10 µs, respectfully.

Total Phosphorylation Calculation

Using the sophisticated numerical annotation procedure (SNAP) peak-picking algorithm, the intensity of individual phosphorylated proteoforms was determined. The total phosphorylation (P_{total}; mol of Pi/mol of protein) for each protein was calculated from those values using the following equation:

$$\boldsymbol{Ptot.} = \frac{(\boldsymbol{intensity\ of\ monophosphorylation\ x1}) + (\boldsymbol{intensity\ of\ bisphosphorylation\ x2})}{\boldsymbol{mols\ of\ total\ protein}}$$

Supplemental Figures

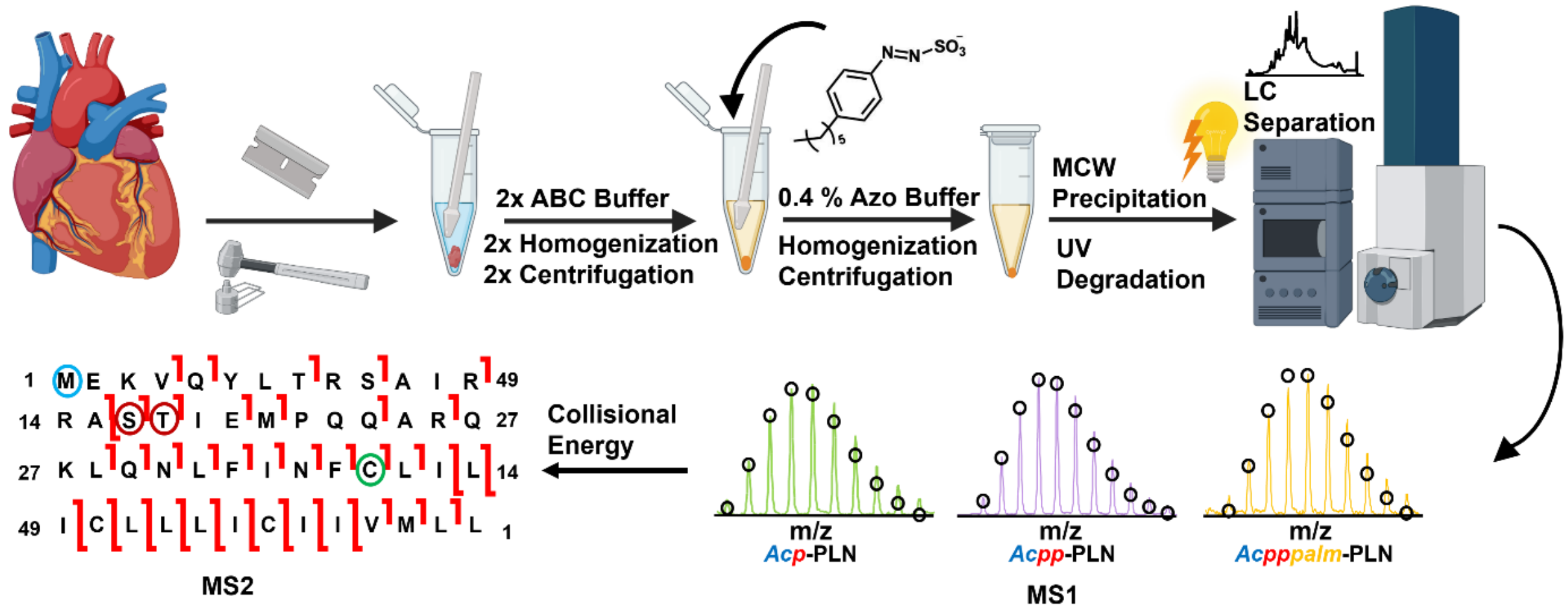

Figure S1. Schematic of the conventional two-stage TDP workflow from 10 mg of cardiac tissue. After being cut and cryopulverized**,** cardiac tissue was homogenized in an ammonium bicarbonate (ABC) buffer twice. Following cytosolic depletion, the pellet was homogenized in Azo buffer, followed by a MCW precipitation. The protein pellets were resolubilized, Azo was degraded by UV irradiation, and intact proteins were analyzed by liquid chromatography–tandem mass spectrometry (LC-MS/MS) using a Bruker Impact II QTOF mass spectrometer.

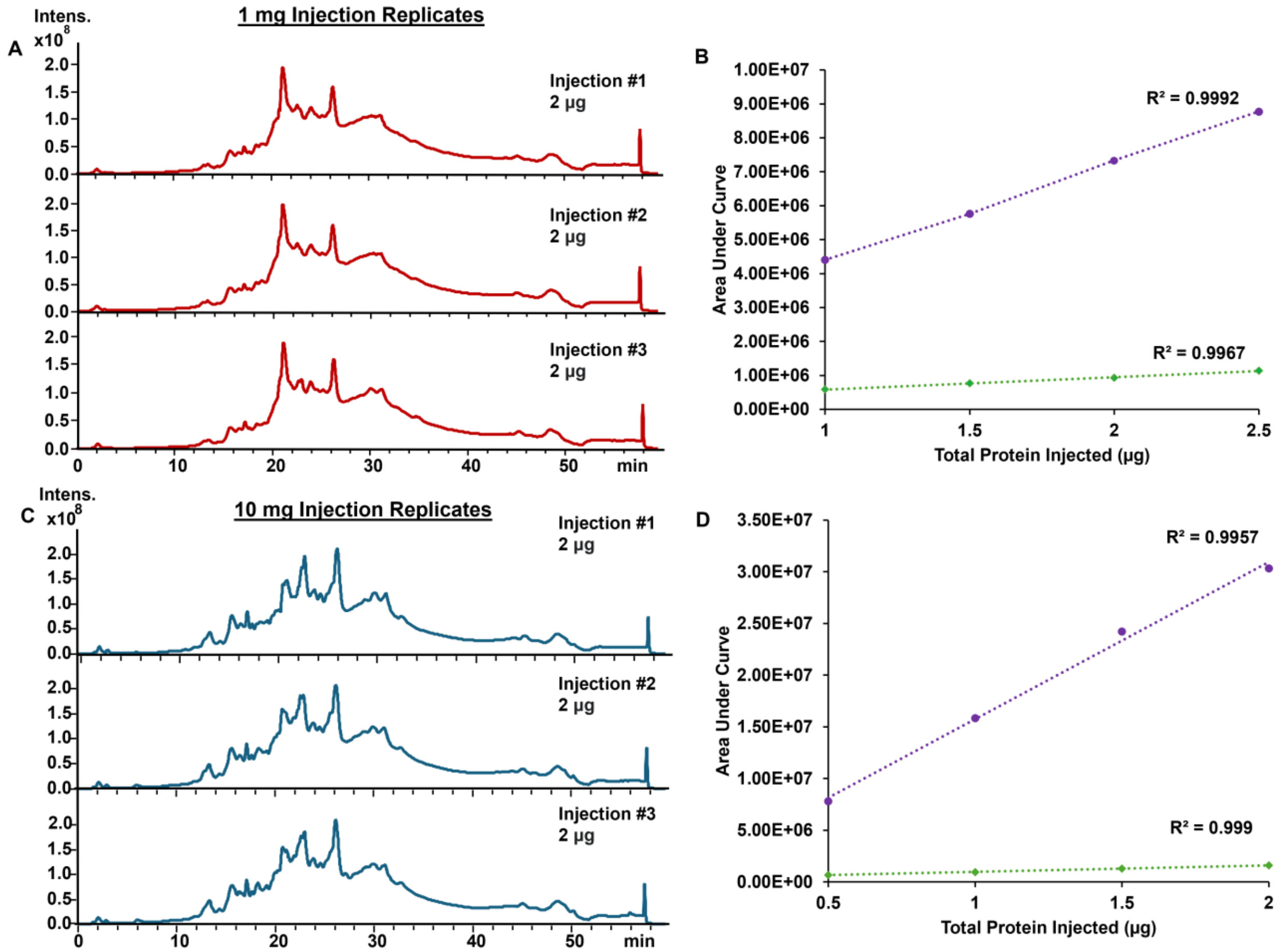

Figure S2. Instrument reproducibility. (**A,B**). Three injections from the same one-pot extraction (1 mg) demonstrate instrument reproducibility (A), and a loading curve from 0.5–2 µg of the one-pot extraction (1 mg) samples injected demonstrate that the amount of sample injected was within the linear range of the instrument (B). (**C,D**). The conventional two-stage extraction (10 mg) replicates demonstrate instrument reproducibility (C), and loading curve from 0.5–2 µg of the conventional two-stage extraction (10 mg) samples injected demonstrate that the amount of sample injected was within the linear range of the instrument (D).

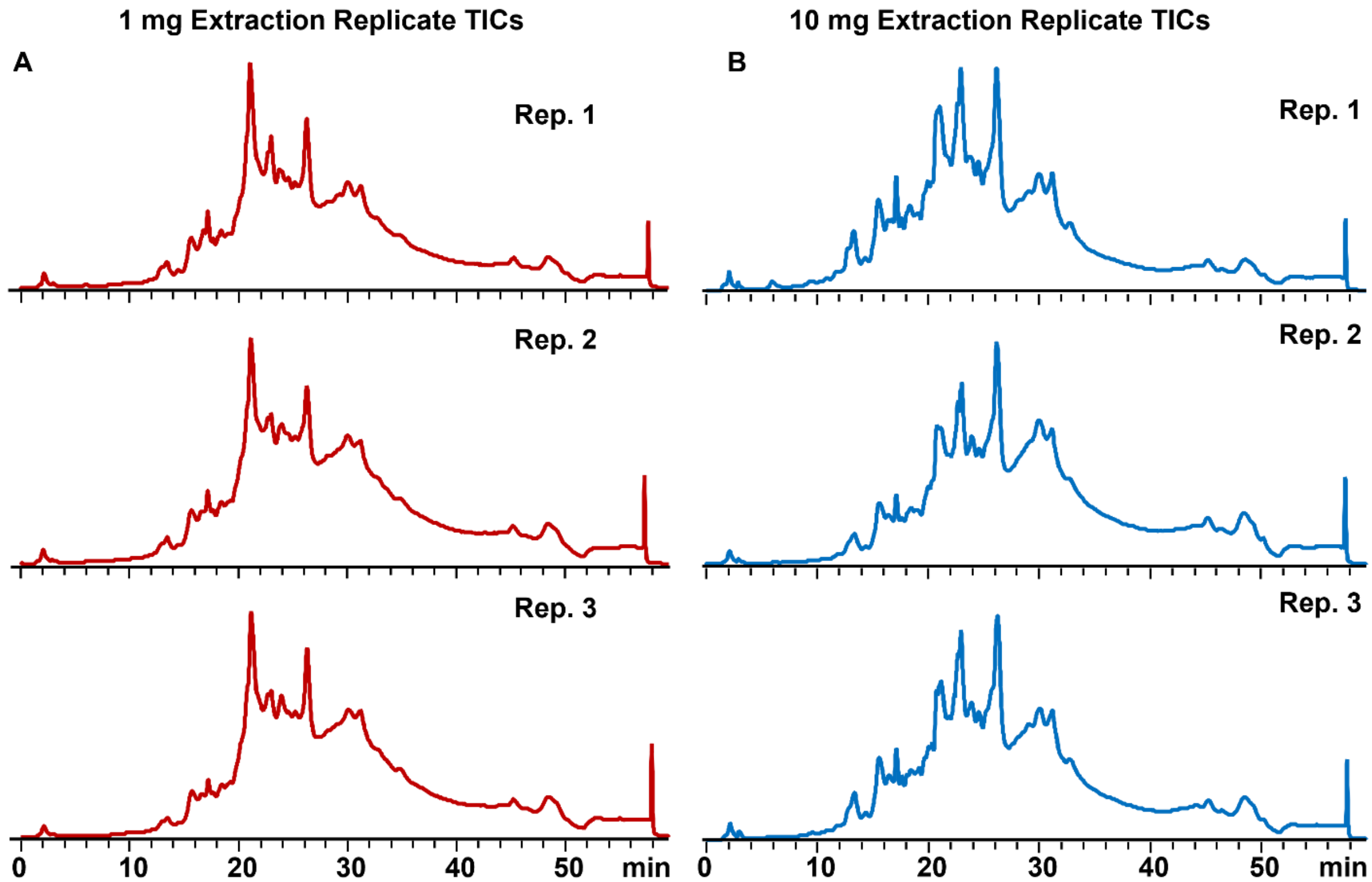

Figure S3. **Total Ion Chromatograms (TIC)s from each extraction replicate**. (**A**) One-pot extraction (1 mg) replicates and (**B**) conventional two-stage extraction (10 mg) replicates demonstrate reproducibility between each method.

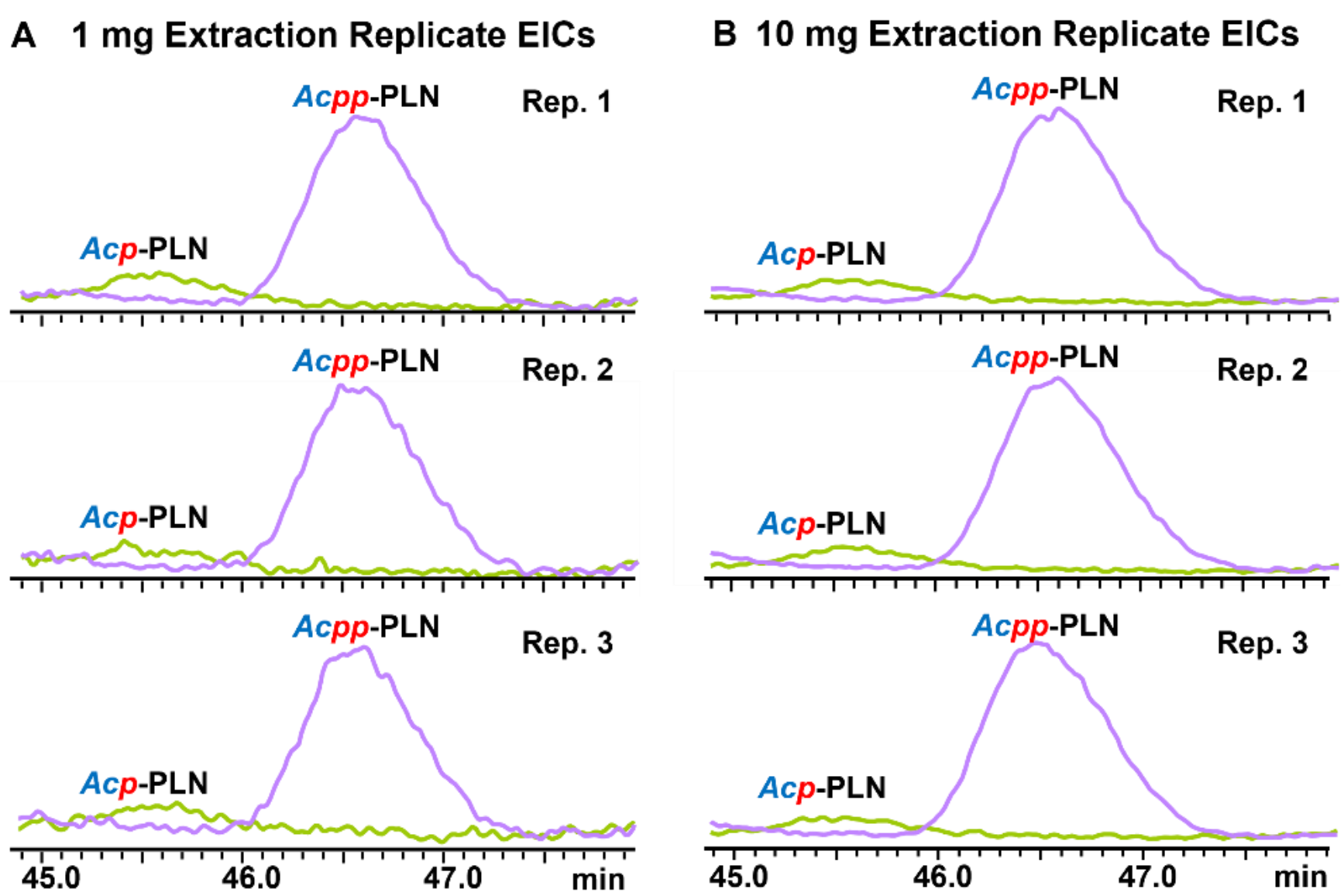

Figure S4. Extracted ion chromatograms (EICs) of individual extraction replicates. (**A**) Replicates from the streamlined one-pot extraction method (1 mg) and (**B**) the conventional two-stage extraction method (10 mg) demonstrate high reproducibility, with PLN proteoforms eluting between 45.0 and 47.5 min.

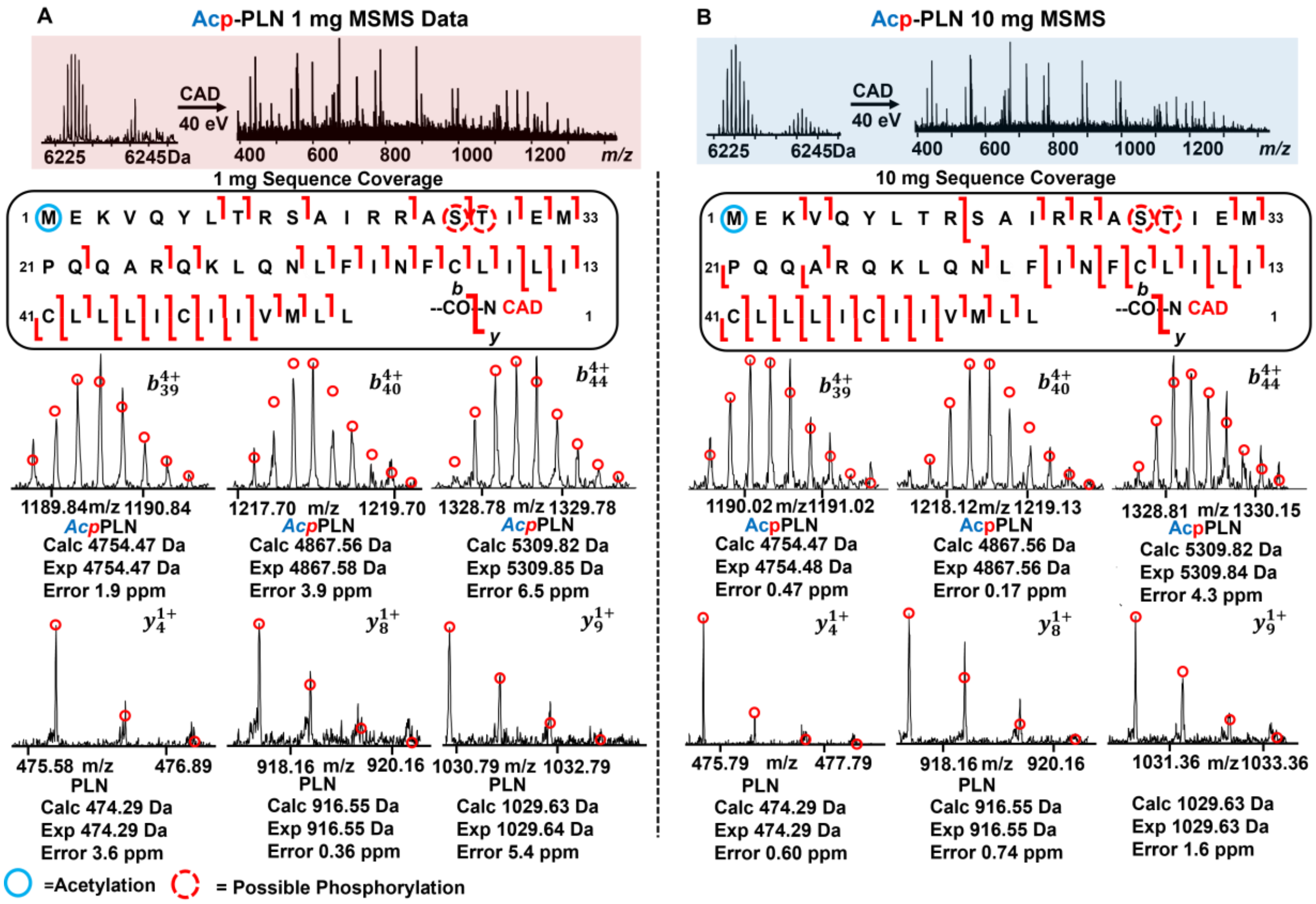

Figure S5. Online LC-MS/MS characterization of the mono-phosphorylated PLN proteoform following the streamlined one-pot (1 mg) and conventional two-stage (10 mg) extraction methods. (**A**) Representative tandem mass spectrum acquired at a collision energy of 40 eV from the 1 mg extract. The sequence map and representative *b*- and *y*-type fragment ions were generated by combining fragment ions from collisional activated dissociation (CAD) spectra acquired at 20, 30, 40, and 50 eV, yielding 43 fragment ions, 33 bond cleavages, and 63.5% sequence coverage. (**B**) Representative tandem mass spectrum acquired at a collision energy of 40 eV from the 10 mg extract. The sequence map and representative *b*- and *y*-type fragment ions were generated by combining fragment ions from CAD spectra acquired at 20, 30, 40, and 50 eV, yielding 47 fragment ions, 32 bond cleavages, and 61.5% sequence coverage.
